# Antibacterial and antioxidant phlorizin-loaded nanofiber film effectively promotes the healing of burn wounds

**DOI:** 10.3389/fbioe.2024.1428988

**Published:** 2024-08-05

**Authors:** Ying Yang, Shuang Ma, Anning Li, Guofeng Xia, Min Li, Chuanbo Ding, Xiaofei Sun, Li Yan, Min Yang, Ting Zhao

**Affiliations:** ^1^ College of Traditional Chinese Medicine, Jilin Agricultural Science and Technology University, Jilin, China; ^2^ College of Traditional Chinese Medicine, Jilin Agricultural University, Changchun, China; ^3^ Jilin Aodong Yanbian Pharmaceutical Co, Ltd., Dunhua, China

**Keywords:** chitosan, phlorizin, nanofiber membrane, scalding wound repair, wound dressing

## Abstract

Burns usually result in damage and loss of skin forming irregular wound wounds. The lack of skin tissue protection makes the wound site highly vulnerable to bacterial infections, hindering the healing process. However, commonly used wound dressings do not readily provide complete coverage of irregular wounds compared to regular wounds. Therefore, there is an urgent need to prepare a wound dressing with high antimicrobial efficacy for the administration of drugs to irregular wounds. In this study, a chitosan (CS)/polyvinylpyrrolidone (PVP) composite nanofiber membrane (CS/PVP/Phlorizin) loaded with root bark glycosides (Phlorizin) was developed using an electrostatic spinning technique. The incorporation of phlorizin, a natural antioxidant, into the fiber membranes notably boosted their antimicrobial and antioxidant capabilities, along with demonstrating excellent hydrophilic characteristics. *In vitro* cellular experiments showed that CS/PVP/Phlorizin increased Hacat cell viability with the presence of better cytocompatibility. In scald wound healing experiments, Phlorizin-loaded nanofibrous membranes significantly promoted re-epithelialization and angiogenesis at the wound site, and reduced the inflammatory response at the wound site. Therefore, the above results indicate that this nanofiber membrane is expected to be an ideal dressing for burn wounds.

## 1 Introduction

Skin, as one of the most important organs of a human being (Yan et al., 2020), is not only resistant to damage from external factors, but also a thermoregulator of the human body, and intact skin tissues can maintain the stability of the internal environment of the human body (Ursell et al., 2012; Liao et al., 2015). Whereas, scald injuries, as one of the most serious skin injuries, are showing an increasing trend in incidence globally (Xu et al., 2020). Burns can be classified based on the depth and extent of skin damage: superficial burns affect only the epidermal layer, partial-thickness burns extend into the dermis, and full-thickness burns penetrate both the epidermal and dermal layers (Wang et al., 2018), and full-layer skin injuries completely rupture the epidermal and dermal layers of the skin. Infection by microorganisms at the burn site contributes to slow recovery of the wound site, increasing the risk of scar formation at the wound site as the healing time increases (Lee et al., 2017). At the same time, scald injuries often result in extremely complicated acute trauma, deformity, disability, or even death, and therefore often cause physical and emotional stress to the scalded patient (Xi et al., 2023). In today’s clinical treatment, topical administration at the wound site is usually used. As well as the use of a variety of traditional dressings to cover the scald wound to avoid infection of the wound site (Guo et al., 2022), such as sponges, bandages, and foam, but due to the adhesive effect of the tissue at the wound site, when changing the dressings often bring secondary damage to the patient (Boateng et al., 2008; Ouyang et al., 2024). Therefore, it is urgent to find a method that is more beneficial for the repair of burn wounds. Compared with traditional excipients, nanofiber membranes are not only simple to prepare, versatile, and easy to replace (Khan et al., 2020), but can also absorb wound site exudate, which is beneficial to the exchange of gases at the wound site and prevents bacterial and microbial infections (Lan et al., 2021; Dong et al., 2023). Due to these beneficial qualities, nanofiber membranes are expected to be a valuable dressing for burn wounds.

Chitosan, a natural polymer derived from chitin (Rinaudo, 2006; Song et al., 2018) has garnered significant attention for its use in the preparation of wound dressings due to its exceptional biocompatibility and various beneficial effects such as antibacterial properties, hemostatic abilities, and promotion of cell proliferation (Archana et al., 2013; Zhou et al., 2016). In addition, chitosan has a broader and more efficient antimicrobial activity than other antimicrobial agents, which enhances its biomedical applications. In recent years, chitosan is often used to prepare nanofiber membrane materials, Wei et al. prepared a CS tetragonal composite with effective control of drug release and antibacterial promotion of infected wound repair (Wei et al., 2024). Yin et al. prepared a CS/PLA nanofiber membrane loaded with aloe-emodin, which exhibited high porosity as well as suitable swelling and hydrophobic properties (Yin et al., 2022). Tang et al. prepared a chitosan/sodium cellulose sulfate composite nanofibrous membrane containing silver nanoparticles, which not only existed excellent antimicrobial properties but also significantly shortened the wound repair time (Tang et al. 2022). Referring to the results of existing studies, it was hypothesized that CS-based nanofiber membrane materials have the potential to be excellent wound dressings. However, due to the rapid degradation of CS materials in acidic environments, their use alone does not meet the needs of ideal wound dressings (Wei et al., 2024), so they are often blended with other polymers such as poly (vinyl alcohol) (PVA), poly (vinylpyrrolidone) (PVP), and so on, to improve the performance of nanomaterials and better meet the needs of wound dressings.

Polyvinylpyrrolidone (PVP) is a spinning polymer with excellent fiber membrane formation properties and is often used as a feedstock for making nanofiber membranes (Poonguzhali et al., 2017). Contardi et al. showed that PVP electrostatically spun hydrogels loaded with hydroxycinnamic acid derivatives significantly reduced the inflammatory response at the wound site, thereby promoting wound site healing (Contardi et al. 2021). Phlorizin, a widely available flavonoid, is an important plant-derived dihydrochalcone (Stompor et al., 2019). Dihydrochalcone as a natural antioxidant has received a lot of attention from scientists in recent years (Li et al., 2018). It has been shown that root bark glycosides can regulate the NF-KB signaling pathway to play an antioxidant role, it also has anti-inflammatory, antiviral, antidiabetic, and other functions (Wang et al., 2019; Un et al., 2021). Sun (Sun et al., 2022)et al. demonstrated that SF/PVP nanofiber membranes containing root bark glycosides reduced inflammation at wound sites by decreasing the expression of TNF-α and IL-1β proteins, thus accelerating wound healing. Additionally, Liu et al. (Liu et al., 2021) found that phlorizin increased levels of superoxide dismutase (SOD) and total antioxidant capacity. However, the use of Phlorizin in scald wound repair has not been reported. Therefore, in this paper, root bark glycosides were loaded into CS/PVP nanofiber membranes to investigate their effects on scald wound repair.

**SCHEME 1:**
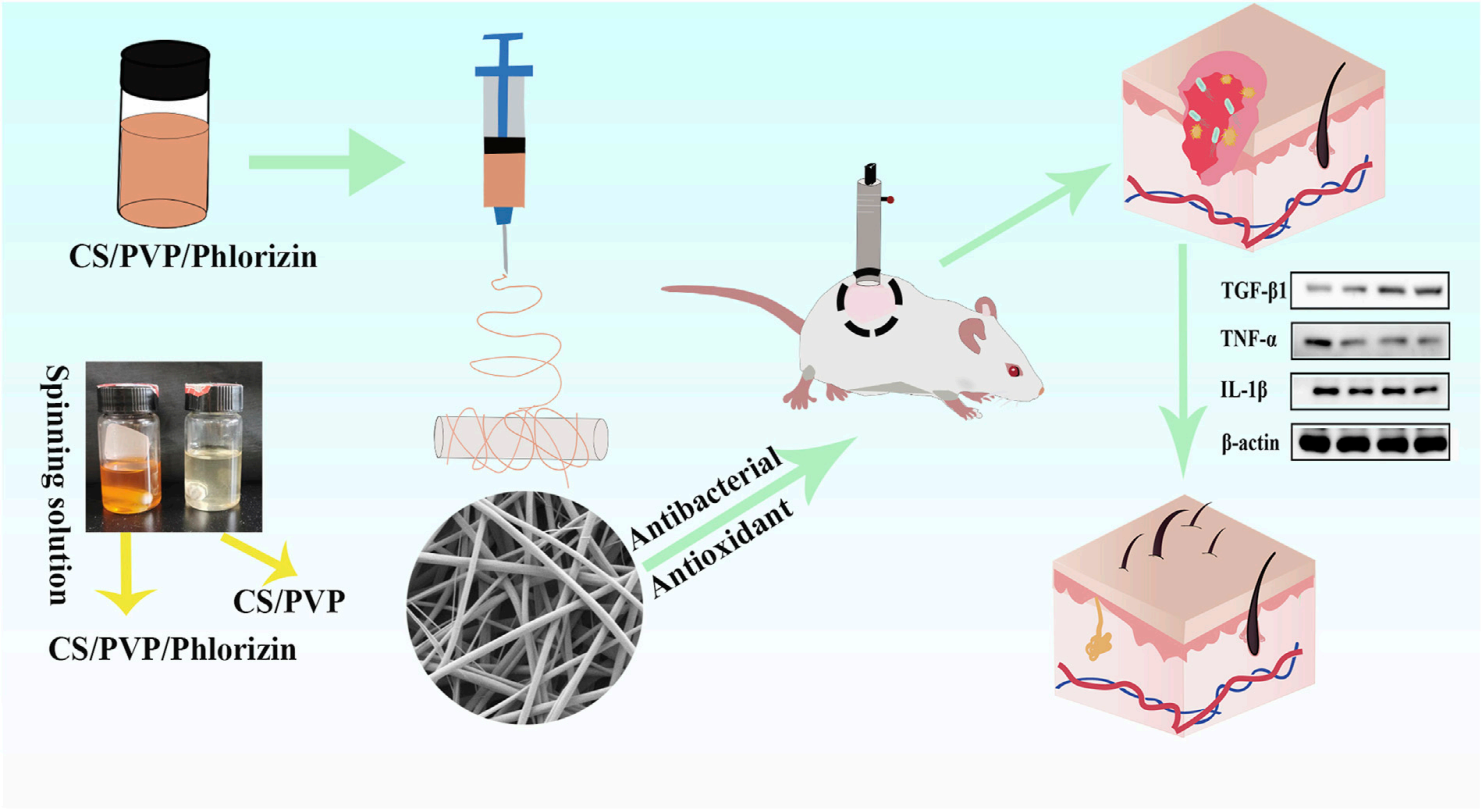
Loaded phlorizin nanofiber membrane to promote scald wound healing

As shown in Scheme 1, in this study, chitosan (CS) and polyvinylpyrrolidone (PVP) were used to synthesize blank fiber membranes by electrostatic spinning technique, in addition to loading phlorizin with antioxidant activity into the blank nanofiber membranes (CS/PVP/Phlorizin). The chemical structure and properties of CS/PVP/Phlorizin were also evaluated by various characterization methods, in addition to their antioxidant and antibacterial activities by *in vitro* antioxidant and antibacterial assays. The biosafety of CS/PVP/Phlorizin was evaluated through MTT cytotoxicity and hemocompatibility tests. And finally, the effect of CS/PVP/Phlorizin nanofiber membrane on scald wound repair was investigated. It is expected that CS/PVP/Phlorizin nanofiber membrane with antioxidant and antibacterial properties will provide a new idea for the treatment of burn wound dressing.

## 2 Experimental section

### 2.1 Experimental materials

Chitosan (CS) was purchased from Sinopharm Chemical Reagent Co, Ltd. Polyvinylpyrrolidone (PVP, Mn = 1.3 MDa). Phlorizin was purchased from Lemaitan Pharmaceuticals Dexter Biotechnology Company Limited (purity ≥80%). Meibao Scald Cream was purchased from Shantou Meibao Pharmaceutical Co. 2,2-Biazobis (3-ethyl-benzothiazole-6-sulfonic acid) diammonium salt (ABTS) was purchased from McLean Biochemicals Ltd. Protein Antibodies (See Support Information Table 1 for details).

**TABLE 1:** Experimental antibody sources and dilution ratios.

Antibodies	Factory owners
TGF-β1 (DILUTION:1:1000–1:5000)	Proteintech group
IL-1β (DILUTION:1:2000–1:10000)	Proteintech group
TNF-α (DILUTION:1:500–1:2000)	Proteintech group
β-actin (DILUTION:1:5000–1:50000)	Proteintech group
HRP coupled secondary antibodies (SA00001−1 1:2000–1:10000/SA00001-21:2000–1:10000)	Proteintech group

### 2.2 Preparation of CS/PVP, CS/PVP/Phlorizin, nanofiber membranes

CS/PVP/Phlorizin nanofiber membranes were prepared by first adjusting the concentration of the materials in the preliminary stage. CS, PVP, and Phlorizin powder were dissolved in 90% glacial acetic acid solution at concentrations of 4.4%, 2.1%and 2% to prepare 10 mL of spinning solution, which was stirred continuously for 12 h at 50°C on a magnetic stirrer to achieve complete dissolution and to obtain a homogeneous spinning solution. Finally, through the electrostatic spinning device, spinning is carried out. The distance from the needle to the drum collector was adjusted to 15 cm, the drum speed was 200 r/min, and the voltage was set to 15 KV to obtain the nanofiber membrane, which was vacuum dried overnight (Zhang et al., 2022; Liu et al., 2024). The CS/PVP nanofiber membrane was prepared in the same way as CS/PVP/Phlorizin but without the addition of phlorizin.

### 2.3 Scanning electron microscopy

The microscopic morphology of CS/PVP, CS/PVP/Phlorizin nanofiber membranes was observed by scanning electron microscopy (ZEISS, EVO/18, Germany). Fixed sample, sprayed with gold, and obtained at an accelerating voltage of 15 kV (Liu et al., 2023). The diameters of 100 fibers were randomly screened in the SEM images, and the calculation of the average diameter of the fiber membrane was performed by ImageJ software.

### 2.4 Fourier infrared spectroscopy

The infrared spectrograms of the nanofiber membranes were determined in the range of 4,000–500 cm^−1^ using a Fourier infrared spectrometer (FITR, SpectrumTwo, Perkinekmer, United States). To determine the loading of Phlorizin in nanofiber membranes.

### 2.5 Water contact angle

To assess the hydrophilic and hydrophobic characteristics of CS/PVP and CS/PVP/Phlorizin nanofiber membranes, the two types of nanofiber membranes were prepared as 2-cm-diameter discs, and then the water contact angle of the nanofiber membranes was determined using water contact angle meter (Dataphysics OCA50, Germany). The average contact angle was determined using ImageJ software.

### 2.6 Water vapor transmission rate

The ideal wound dressing should have an appropriate water vapor transmission rate to control the evaporation of water from the wound site (Xia et al., 2020). Referring to the Li method with slight modification (Li et al., 2024), firstly, 5 mL of distilled water was added to a vial with a mouth area of S. Immediately, the circular nanofiber membrane was placed in the mouth of the vial and weighed and recorded as W_i_. They were placed in an incubator at 37°C for 24 h and weighed and recorded as W_t,_ three times in parallel for each group. The water vapor transmission rate was calculated according to the following equation:

$$WVTR\left({gm}^{-2}d^{-1}\right)=\left(Wi-Wt\right)/S$$ (1)

### 2.7 Antioxidant effect

A slight modification was made with reference to the existing method. To determine the scavenging effect of CS/PVP and CS/PVP/Phlorizin nanofiber membranes on ABTS free radicals. Configure 7.5 mM of ABTS solution and 2.45 mM of potassium persulfate solution. The reaction was carried out in a 1:1 ratio for 12 h at room temperature. The working solution was diluted at room temperature until its absorbance at 745 nm was 0.7 ± 0.2. Weighing 5 mg of nanofiber membrane was co-incubated with 2 mL of ABTS solution for half an hour, and the group without fiber membrane was used as a blank control group. The ABTS free clearance was calculated according to the following equation (Ma et al., 2024).

$$Removal\;rate\left(\%\right)=\left(1-A_{s}/A_{c}\right)\times 100\%$$ (2)

As is the absorbance of the fiber membrane plus reaction solution, and Ac is the absorbance of the control without the addition of the nanofiber membrane.

### 2.8 Hemolytic effect

*In vitro*, the blood safety properties of nanofiber membranes were evaluated using hemolysis of mouse erythrocytes. Mouse erythrocytes were first obtained by centrifugation of fresh mouse blood, and saline was chosen to dilute the erythrocyte suspension to 2%. The CS/PVP and CS/PVP/Phlorizin nanofiber membranes were then cut into equal volume (1 cm × 1 cm) discs. An equal volume of nanofiber membrane was taken and co-incubated with 1 mL of 2% erythrocyte suspension at 37°C for 1 h. The 1% Trion X-100 and PBS groups were selected as positive and negative controls. At the end of the incubation, the supernatant was obtained and its absorbance at 545 nm was determined and calculated according to the following formulae (Lian et al., 2021).

$$Hemolysis\;rate\left(\%\right)=\left(A_{S}-A_{P}\right)/\left(A_{y}-A_{p}\right)\times 100\%$$ (3)

Among them, A_s_ is the sample group, A_p_ is the negative control group, and A_y_ is the positive control group.

### 2.9 Cytotoxicity

Cytotoxicity of CS/PVP and CS/PVP/Phlorizin membranes was determined using Hacat skin keratinocytes. The CS/PVP, CS/PVP/Phlorizin nanofiber membranes were first sterilized by UV irradiation for 2 h. The sterilized nanofiber membrane was then dissolved in the medium to obtain a drug-loaded medium. Hacat cells in logarithmic growth phase were taken and inoculated at a density of 2 × 10^4^/well in 96-well plates in an incubator at 37°C, 5% CO_2_ for 24 h, and then replaced with drug-loaded medium. In the control group, the complete medium was replaced and the culture was continued for 24 h, then 20 μL of MTT solution was added to each well and reacted for 4 h, 150 μL of DMSO was added to each well sequentially, and the absorbance was measured at 490 nm to calculate the cell viability of each group.

### 2.10 Bacteriostatic effect

*Staphylococcus aureus* and *Escherichia coli* were selected as model bacteria to evaluate the *in vitro* antimicrobial activity of nanofiber membranes (CS/PVP, CS/PVP/Phlorizin) by colony counting method. Firstly, the nanofiber membrane with a diameter of 1 cm was cut and sterilized by irradiation under UV lamp for 2 h, and the fiber membrane was co-cultivated with the bacterial solution (37°C, 2 h). Absorb 30 μL of the above bacterial solution evenly on solid medium agar plate, without soaking fiber membrane bacterial solution as a control group, and incubate at 37°C for 12–24 h. The bacterial inhibition rate of the nanofiber membrane was calculated according to the following equation (Li et al., 2022).

$$Bacterial\;inhibition\;rate\left(\%\right)=\frac{N_{c}-N_{s}}{N_{c}}\times 100\%$$ (4)

N_c_ is the control group and N_s_ is the number of colonies in the sample group.

### 2.11 *In vivo* scald wound repair

To evaluate the effect of CS/PVP/Phlorizin nanofiber membranes on scald wounds, this paper first creates a scald wound model, which complies with the National Research Council Guide for the Care and Use of Laboratory Animals. In this paper, ICR male mice (25 ± 5 g) were purchased from Changchun Yisi Laboratory Animal Technology Co, mice were first acclimatized for 1 week after rearing. Mice were anesthetized using 4% chloral hydrate and depilatory cream was applied for hair removal. Using the YLS-5Q scalding instrument, a 2.5-cm^2^ metal punch was selected, and the temperature was set to 85°C with a pressure of 500 KPa pressed tightly to the hair removal site of the mice for 10 s, to construct a scalding wound (Jieqiong, 2020). Cage hygiene was strictly controlled after molding was completed and an adequate diet was provided. The scalded mice were randomly divided into four groups of 10 mice each, namely, the scald model group, the positive control group, the CS/PVP, and the CS/PVP/Phlorizin groups, with no treatment in the model group, and the other three groups were given daily treatment. Photographs were taken on days 3, 7, 14, and 21 after treatment to observe changes in the wound site, i.e., changes in the color of the wound site, whether it was infected or not, scabbing, and changes in the size of the wound. The wound area was calculated using ImageJ software. The healing rate of the wound area was calculated according to the following formula (Asiri et al., 2021).

$$Wound\;healing\;rate\left(\%\right)=\frac{S_{o}-S_{t}}{S_{o}}\times 100\%$$ (5)

S_o_ is the initial wound area and S_t_ is the fixed-time wound area.

### 2.12 H&E and Masson staining

For further observation of tissue changes and collagen deposition at the wound site. Skin tissues from each group of mice were taken at the fixation time, and after fixation with 4% paraformaldehyde, the residual fixative was removed from the skin tissues using water. Then it was gradient dehydrated with ethanol, cleared with xylene, paraffin embedded, and then cut into 6um sections for H&E and Masson staining.

### 2.13 Immunohistochemical staining

On day 21 of constructing the scald wounds, skin tissues from the wound site were taken for paraffin embedding, sectioned, and then subjected to immunohistochemical analysis. Firstly, the paraffin sections were deparaffinized and washed with PBS, and 3% BSA was chosen to be closed for half an hour. Then α-SMA, CD31, and VEGF primary antibody were diluted according to the proportion, and incubation was carried out. After incubation for 50 min at room temperature with 2 antibodies corresponding to the primary antibody, the sections were finally stained and observed by microscope.

### 2.14 Western blot

Skin tissues from the wound site of scalded mice in each group were taken and weighed, and proteins in the skin tissues of each group were extracted by adding RIPA lysate containing PMSF. The protein content in each group of samples was first determined by the BCA method. Sample proteins were electrophoresed through sodium dodecyl sulfate polyacrylamide gel (10%). After waiting for the end of electrophoresis, the membrane was rotated at low temperature for 90 min under the condition of constant voltage 100 V. The PVDF membrane was selected to be closed with 5% skim milk powder for 2 h. The membrane was incubated with the specific primary antibody for 1.5 h. At the end of the incubation, the membrane was washed with TBST 3 times for 10 min each time. Then the membrane was incubated with HPR-labeled secondary antibody for 1 h. The membrane was washed three times in TBST for 10 min each finally, the protein expression level was detected by gel imaging analysis system.

### 2.15 Statistical analysis

All experimental data were repeated at least three times and expressed as mean ± standard deviation (SD). One-way analysis of variance (ANOVA) was performed using SPSS 22.0 software, and a value of *p < 0.05 indicates a significant difference.

## 3 Results

### 3.1 Scanning electron microscopy

**FIGURE 1:**
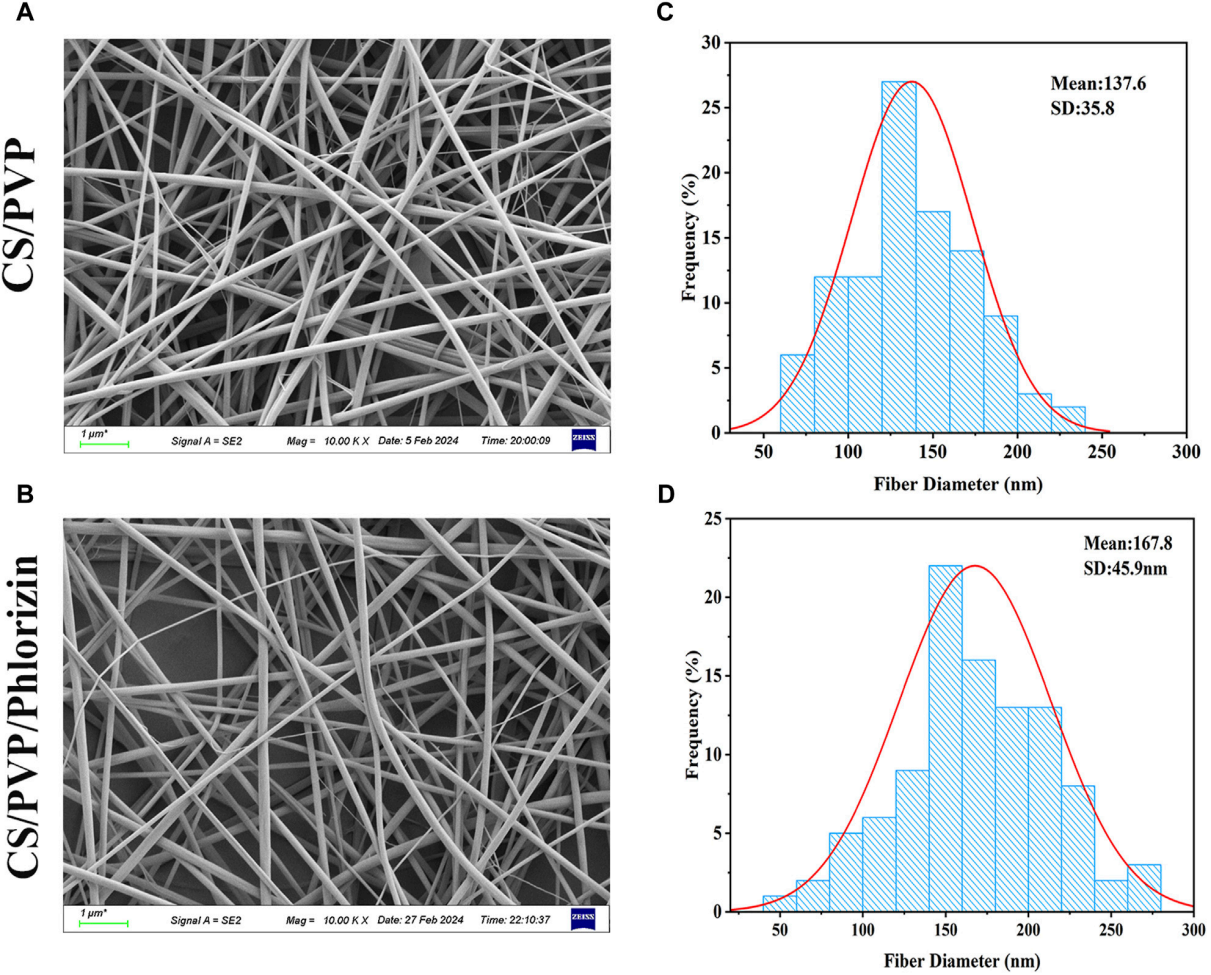
SEM image of nanofiber membrane and its nanofiber diameter size. **(A, B)** SEM images of CS/PVP, CS/PVP/Phlorizin nanofiber membranes; **(C, D)** Diameter distribution of CS/PVP, CS/PVP/Phlorizin nanofiber membranes, results were used as mean ± standard deviation.

The microscopic morphology and diameter of CS/PVP and CS/PVP/Phlorizin nanofiber membranes are shown in Figure 1. As shown in Figure 1A, B it can be observed that the nanofibers in the two types of fibrous membranes exist crosswise and twisted with each other, thus forming a porous three-dimensional structure. Due to this structure, nanofiber membranes are beneficial for the exchange of gases at the wound site as well as the transport of nutrients and the growth and migration of cells at the wound site (Hasani-Sadrabadi et al., 2019). The average diameters of CS/PVP and CS/PVP/Phlorizin were 137.6 ± 35.8 nm and 167.8 ± 45.9 nm, respectively. It was also found that the diameter of the nanofibers increased with the loading of Phlorizin, and at the same time, no non-homogeneous structure was observed on the surface of the nanofibers, indicating that the Phlorizin was fully dissolved in the spinning solution.

### 3.2 Fourier infrared spectroscopy

**FIGURE 2:**
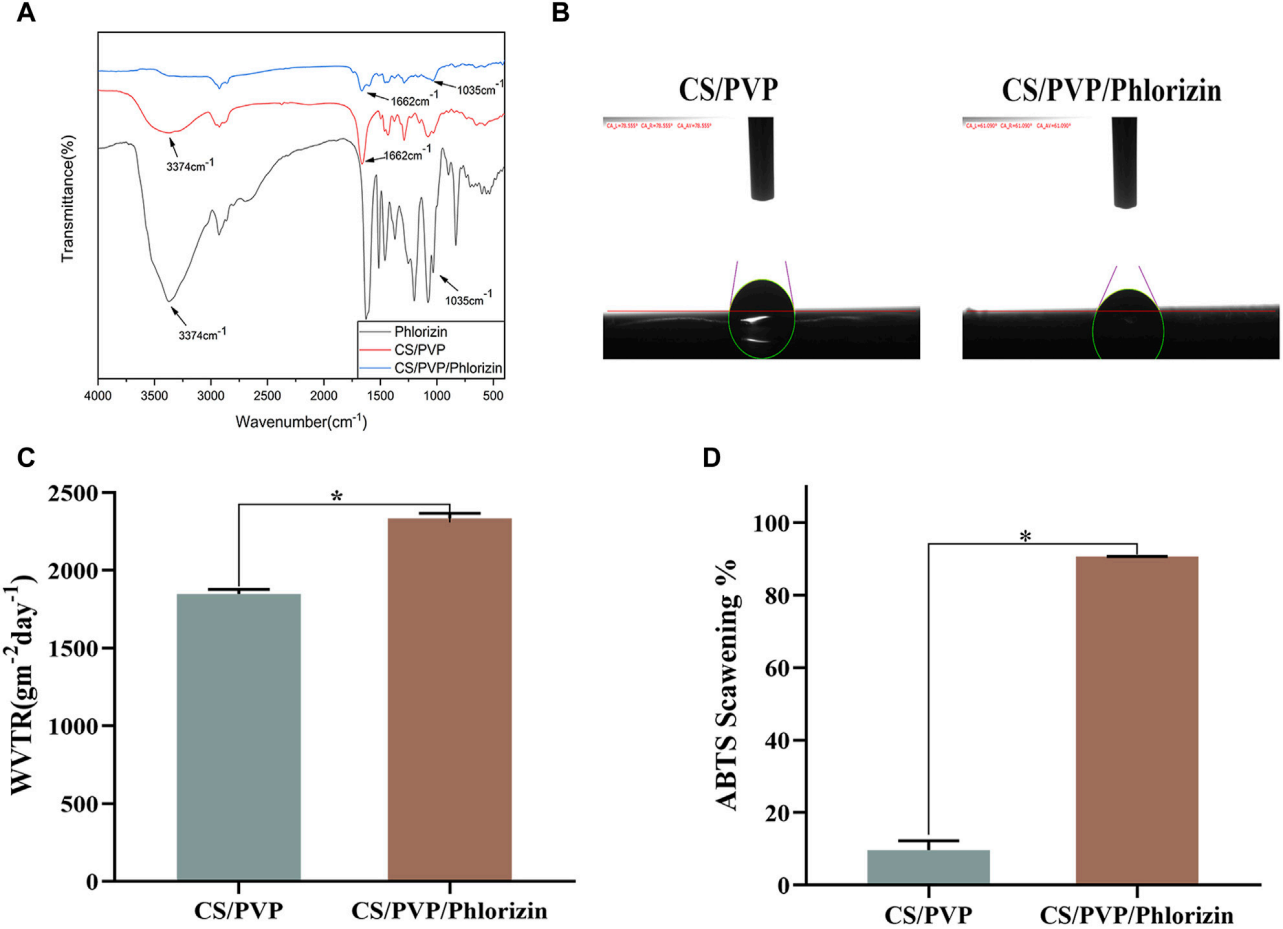
**(A)** Fourier infrared spectra; **(B)** water contact angle; **(C)** water vapor transmission rate; **(D)** ABTS radical scavenging rate.

The infrared scanning results are shown in Figure 2A. The characteristic peak at 3,374 cm^−1^ corresponds to the O-H tensile vibration. Both CS/PVP and CS/PVP/Phlorizin nanofiber membranes display a characteristic peak of the amide I bond at 1,662 cm^−1^, associated with C=O stretching vibration. The waveform of the CS/PVP/Phlorizin scaffold amide I bond broadened upon loading of Phlorizin. The CS/PVP/Phlorizin nanofibers exhibited a characteristic C-O peak at 1,035 cm^−1^, which is the characteristic absorption peak of Phlorizin.

### 3.3 Water contact angle

Nanofiber membrane materials can quickly absorb excess exudate from the wound site, thus reducing the wound infection rate, while providing a moist microenvironment for the wound site, effectively promoting the proliferation and migration of cells in the wound site thus accelerating the repair of the wound site (Dong and Guo, 2021). In this paper, the hydrophilicity of the surface of the nanofiber membrane was evaluated by determining its water contact angle (He et al., 2016). When the water contact angle of the fiber membrane is less than 90° indicates the existence of good hydrophilicity of the fiber membrane. The results are shown in Figure 2B, and the water contact angles of CS/PVP and CS/PVP/Phlorizin are 78.55° and 61.09°, respectively, indicating the existence of better hydrophilicity of the two materials. Also from the results, it can be seen that the hydrophilicity of the fiber membrane was enhanced after loading phlorizin.

### 3.4 Water vapor transmission rate

Water vapor transmission rate, as one of the most important evaluation indexes of wound dressings, can reflect the gas transmission rate when nanomaterials are applied to the wound site. Compared with the blank fiber membrane, the transmittance of the nanofiber membrane loaded with phlorizin increased, reaching 2334.28 ± 32.16 gm^−2^d^−1^. The water vapor transmission rate of the nanofiber membrane was in the range of 2000–2,500 gm^−2^d^−1^, which meets the water vapor transmission rate requirement for wound healing, and the results are shown in Figure 2C.

### 3.5 Antioxidant activity

Excess free radicals may lead to cell destruction and DNA damage, which can lead to a number of diseases. Therefore, in this paper, the scavenging effects of CS/PVP, and CS/PVP/Phlorizin on ABTS radicals were determined. The results, as shown in Figure 2D, showed that the clearance of CS/PVP, and CS/PVP/Phlorizin for ABTS was 9.6% ± 2.58% and 90.68% ± 0.05%, respectively. The antioxidant activity of the fiber membranes loaded with phlorizin was significantly higher (*p < 0.05) compared with that of the blank membranes, which may be attributed to the good antioxidant activity of phlorizin itself.

### 3.6 Blood compatibility

**FIGURE 3:**
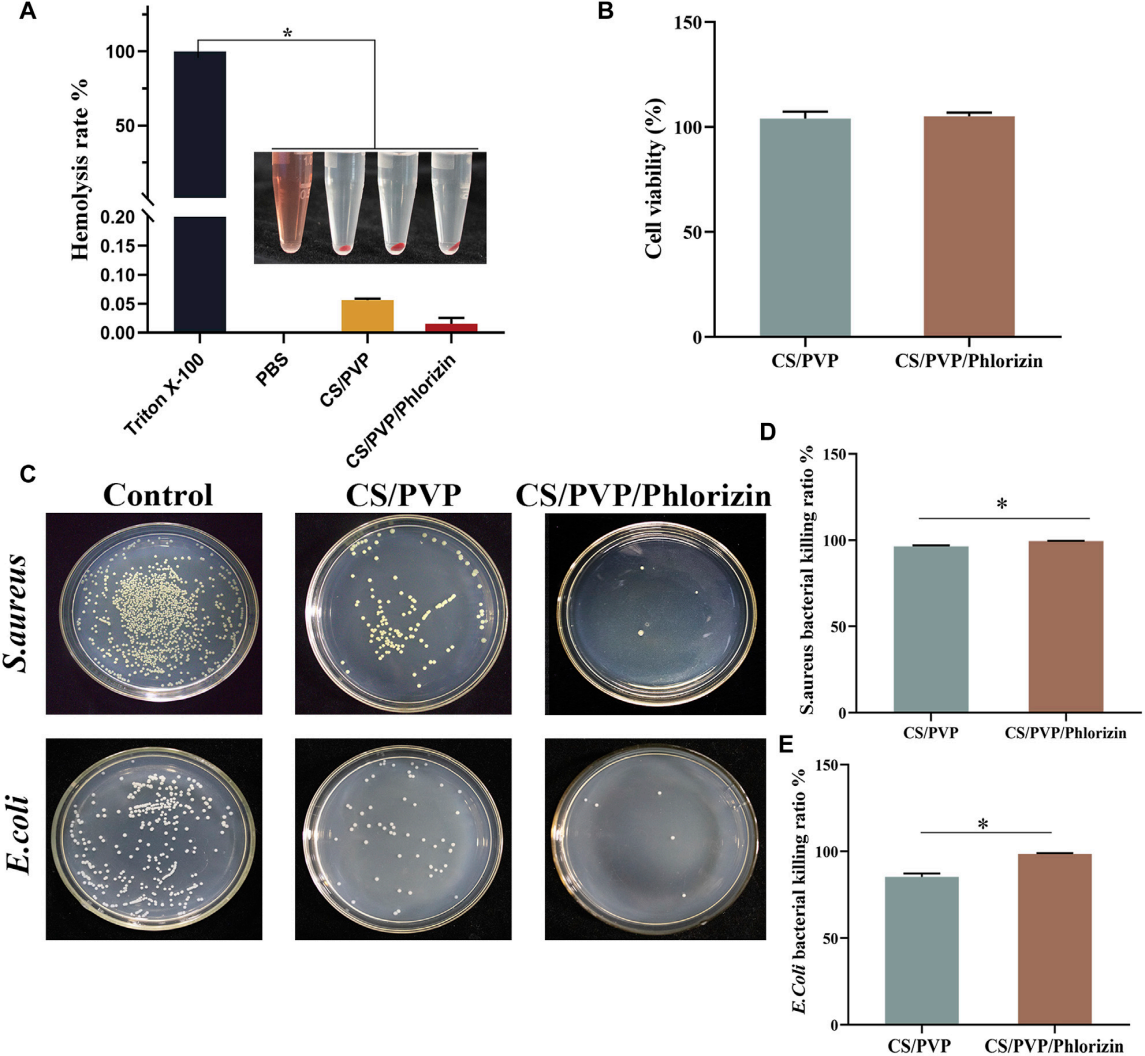
**(A)** Hemocompatibility; **(B)** Cytotoxicity; **(C)** Colony counts of CS/PVP, CS/PVP/Phlorizin nanofiber membranes for *Staphylococcus aureus* and *Escherichia coli*; **(D)** Quantitative graph of the inhibition rate of the nanofiber membranes for *Staphylococcus aureus*; **(E)** Quantitative graph of the inhibition rate of the nanofiber membranes for *Escherichia coli.*

As shown in Figure 3A, the hemolysis rates of CS/PVP and CS/PVP/Phlorizin nanofiber membranes were 0.056% ± 0.026% and 0.016% ± 0.01%, respectively, and the hemolysis rates of both materials were less than 5%. At the same time, it can be observed that the supernatant of two groups of nanofiber membrane groups is clear and transparent, and there is a significant difference from the red colour of the positive control (*p < 0.05). It shows that there is better blood compatibility between the two materials and no hemolysis phenomenon. They meet the basic requirements of safe wound dressing.

### 3.7 Cytotoxicity analysis

Having good biosafety is a fundamental condition for the application of wound dressings. In this paper, the biosafety properties of the two materials were verified by MTT experiments. Skin keratinocytes (Hacat) were incubated with nanofiber membrane extracts for 24 h and all cell viability was greater than 100%. The results of the statistical analysis are shown in Figure 3B, and there was no significant difference between the two groups. It indicates that the two nanofiber membranes exist better cytocompatibility and can promote the proliferation of Hacat cells.

### 3.8 Bacteriostatic activity analysis

During the wound healing process, it is highly susceptible to *E. coli* and *S. aureus*, which can cause tissue infection and inflammation to occur at the wound site, resulting in slow healing of the wound site. Therefore, good bacteriostatic activity is necessary for an ideal wound dressing. The results are shown in Figure 3C, CS/PVP and CS/PVP/Phlorizin can significantly reduce the colony count of *E. coli* and *S. aureus*. The results of the quantitative analysis are shown in Figure 3D, E, the inhibition rates of CS/PVP/Phlorizin were 99.5% ± 0.12% and 98.46% ± 0.47% for *S. aureus* and *E. coli*, respectively. The bacterial inhibition rate of nanofiber membranes loaded with Phlorizin was significantly higher (p* <0.05) compared with that of CS/PVP nanofiber membranes.

### 3.9 *In vivo* mouse scald wound repair

To investigate the role of nanofiber membrane for the repair of scald wounds. We first successfully constructed deep second-degree scald wounds. Macroscopic observation showed that the skin at the scalded site was wrinkled and yellowish-white, and the skin at the scalded site was hardened when lightly touched, and the pain sensation of the mice was significantly increased. Indicates successful modeling of deep second-degree burns.

**FIGURE 4:**
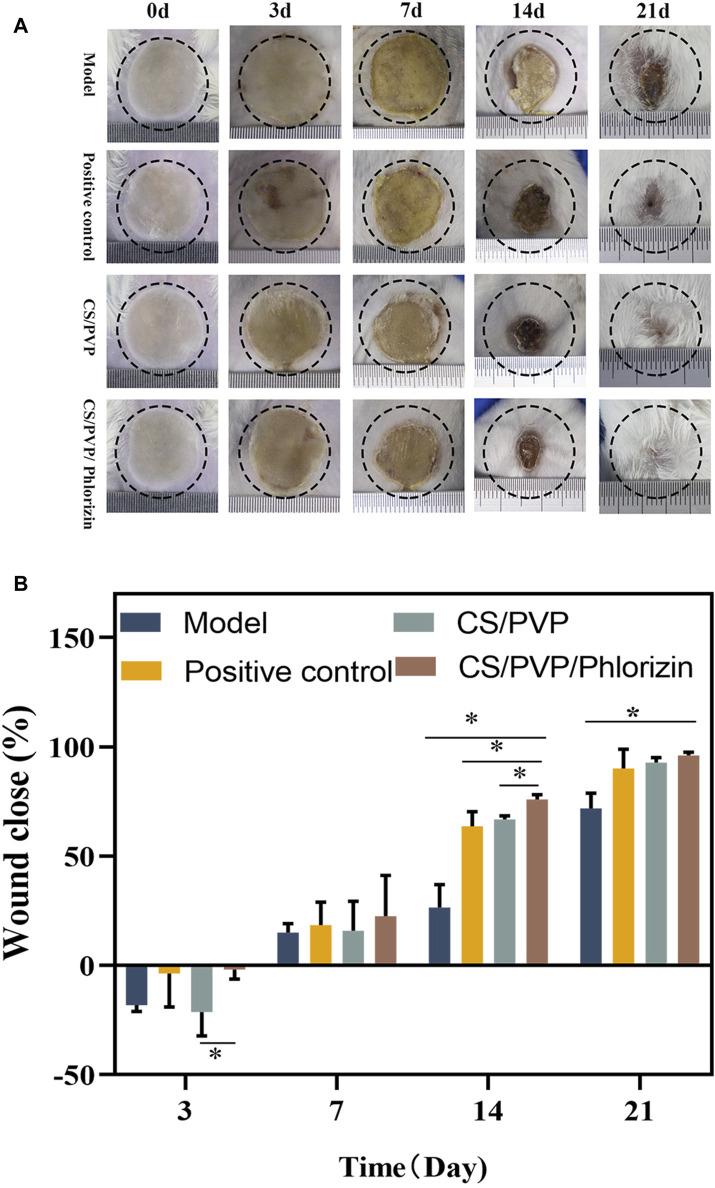
**(A)** Wound status and its size change on days 0, 3, 7, 4, and 21 for Model, Positive control, CS/PVP, and CS/PVP/Phlorizin groups; **(B)** Quantitative plot of wound close rate.

The state of the skin at the site of the wound was also observed, and the degree of wound closure was evaluated to assess the healing of the scald wounds in each group, and the results are shown in Figure 4A. On the third day of constructing the scald wounds, the relative enlargement of the wounds in each group may be attributed to the inflammatory response at the wound site that enlarged the wound area, which is consistent with what has been reported. At day 7 of scald wound repair, a thick scab was present on the wound surface in all groups, but partial shedding of the crust was visible in the CS/PVP and CS/PVP/Phlorizin nanofibrous membrane groups. On the 14 days after treatment, the scabs were completely removed from the wound site in the remaining three groups compared to the model group. A smaller wound area existed in the rhizoposide-loaded nanofiber membrane group compared to the CS/PVP nanofiber membrane group. On the last day of wound healing, the wounds of the phlorizin loaded nanofiber membrane group were completely closed, which was significantly different from the wounds of the model group. It indicates that the nanofiber membrane loaded with phlorizin can significantly promote the wound repair of scalded wounds.

### 3.10 Histopathologic analysis

**FIGURE 5:**
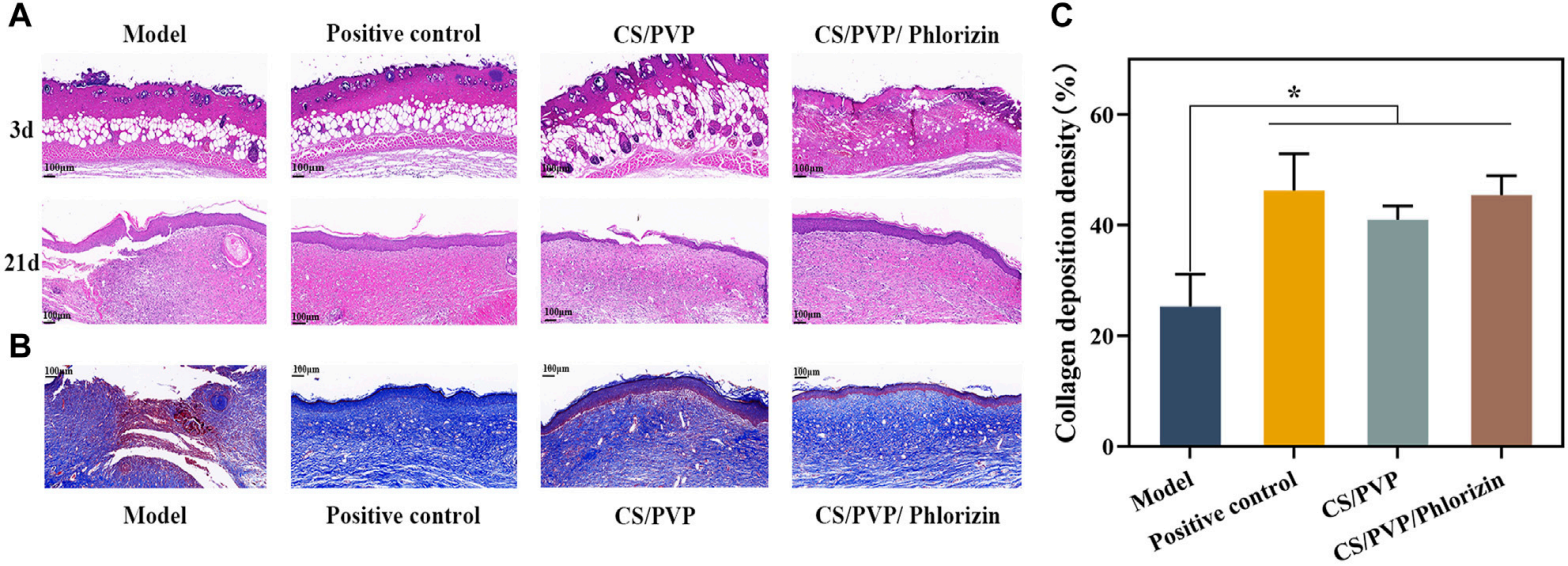
**(A)** H&E staining of Model, Positive control, CS/PVP, and CS/PVP/Phlorizin groups on days 3 and 21; **(B)** Masson staining of each group on day 21; **(C)** collagen deposition statistics of each group.

The efficacy of nanofibrous membranes for scald wound healing was evaluated by hematoxylin eosin staining for pathological observation of scald wound site tissues in each group. As shown in Figure 5A the epidermal defects and their subdermal blood vessels and tissues were damaged and necrotic tissues were present in all groups of mice as seen on day 3 of wound creation. Indicates successful creation of a deep second-degree burn wound model. At day 21 of wound healing, the positive control and CS/PVP/Phlorizin groups had been completely re-epithelialized compared to the model group. The CS/PVP/Phlorizin group exhibited thicker granulation tissue and angiogenesis. Suggesting that nanofiber membranes loaded with phlorizin can effectively promote scald wound healing. This may be attributed to the excellent antibacterial and antioxidant effects of the nanofiber membrane, which reduces the rate of infection at the wound site and provides a healing-friendly environment for the wound site (Guo et al., 2020).

The collagen expression (blue color) of each group after 21 days of treating the wounds was observed by Masson staining, and the results are shown in Figure 5B. Protein expression of collagen was significantly higher in the other three groups compared to the model group. Compared to the CS/PVP group, CS/PVP/Phlorizin collagen content was increased and collagen fibers were more compactly arranged in bands (Johnson, 2003). In addition, collagen fibers with a tight structure have been reported to be beneficial for ECM formation, thereby accelerating wound healing (Li et al., 2020).

### 3.11 Immunohistochemical analysis

**FIGURE 6:**
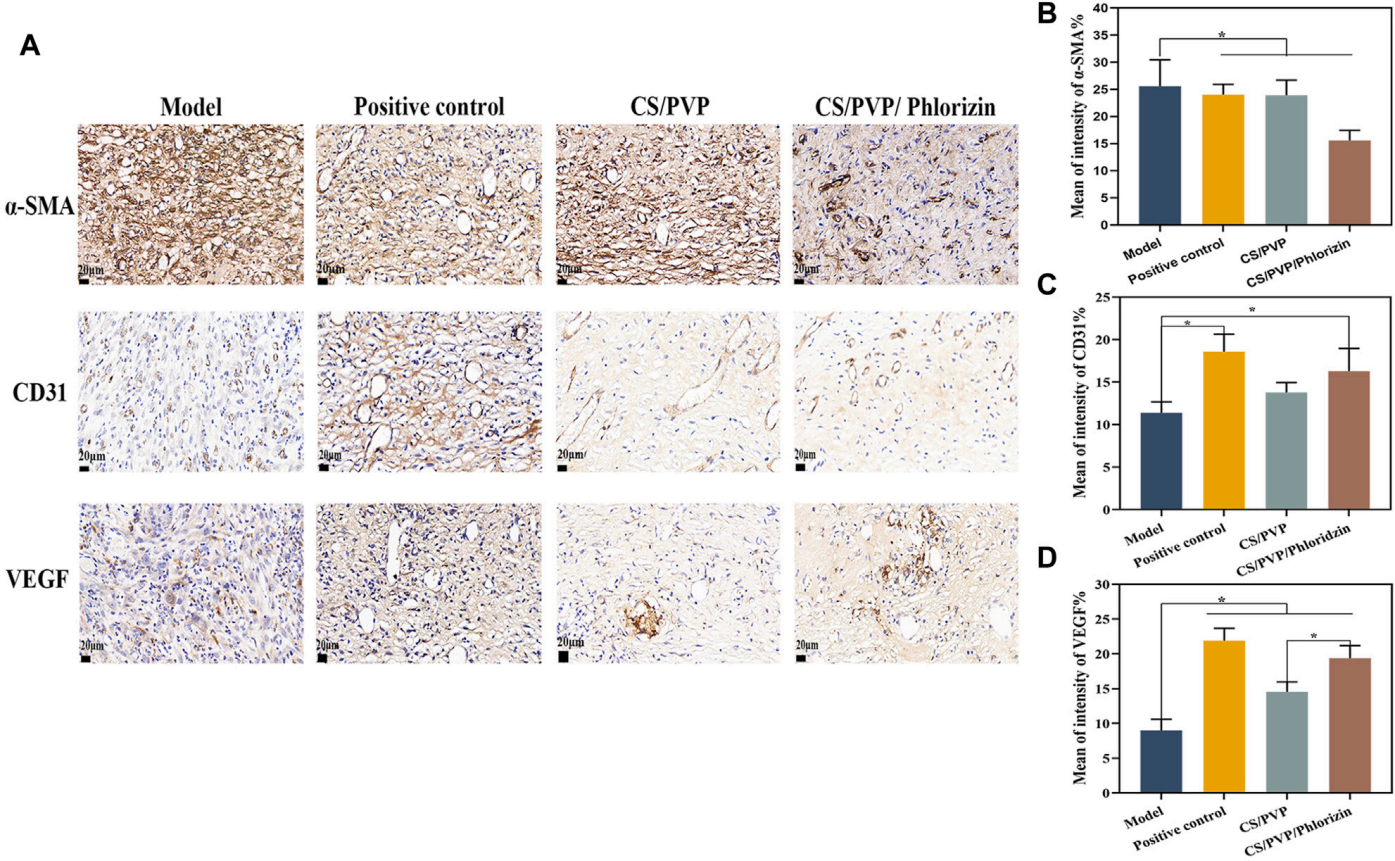
**(A)** Immunohistochemical pictures of α-SMA, CD31, and VEGF in each group; **(B)** Quantitative analysis pictures of α-SMA; **(C)** Quantitative analysis pictures of CD31; **(D)** Quantitative analysis pictures of VEGF.

To further investigate the possible mechanism of CS/PVP/Phlorizin nanofiber membrane to promote the healing of scald wounds, the expression of α-smooth muscle actin (α-SMA), CD31 (early vascular growth factor), and VEGF (vascular endothelial generating factor) was measured at the wound sites of each group. The expression of α-SMA is associated with myofibroblasts, which have a critical role in rapid wound healing, however, rapid contraction at the wound site can lead to scar formation at the wound site (Zonari et al., 2015). The expression of α-SMA was significantly reduced in the CS/PVP/Phlorizin group after 21 days of treatment compared with the model and positive control groups. This result suggests that the nanofiber membrane loaded with phlorizin reduces scar formation at the scald wound site. CD31 and VEGF as vascular growth factors its can respond to the vascular expression at the wound site. Compared to the scald model group, the other three groups showed significantly higher vascular expression (Figure 6A). Higher vascular expression existed in the CS/PVP/Phlorizin group compared to the CS/PVP group, and the results suggest that CS/PVP/Phlorizin nanofibrous membranes promote angiogenesis at the wound site and thus promote wound repair.

### 3.12 Western blot analysis

**FIGURE 7:**
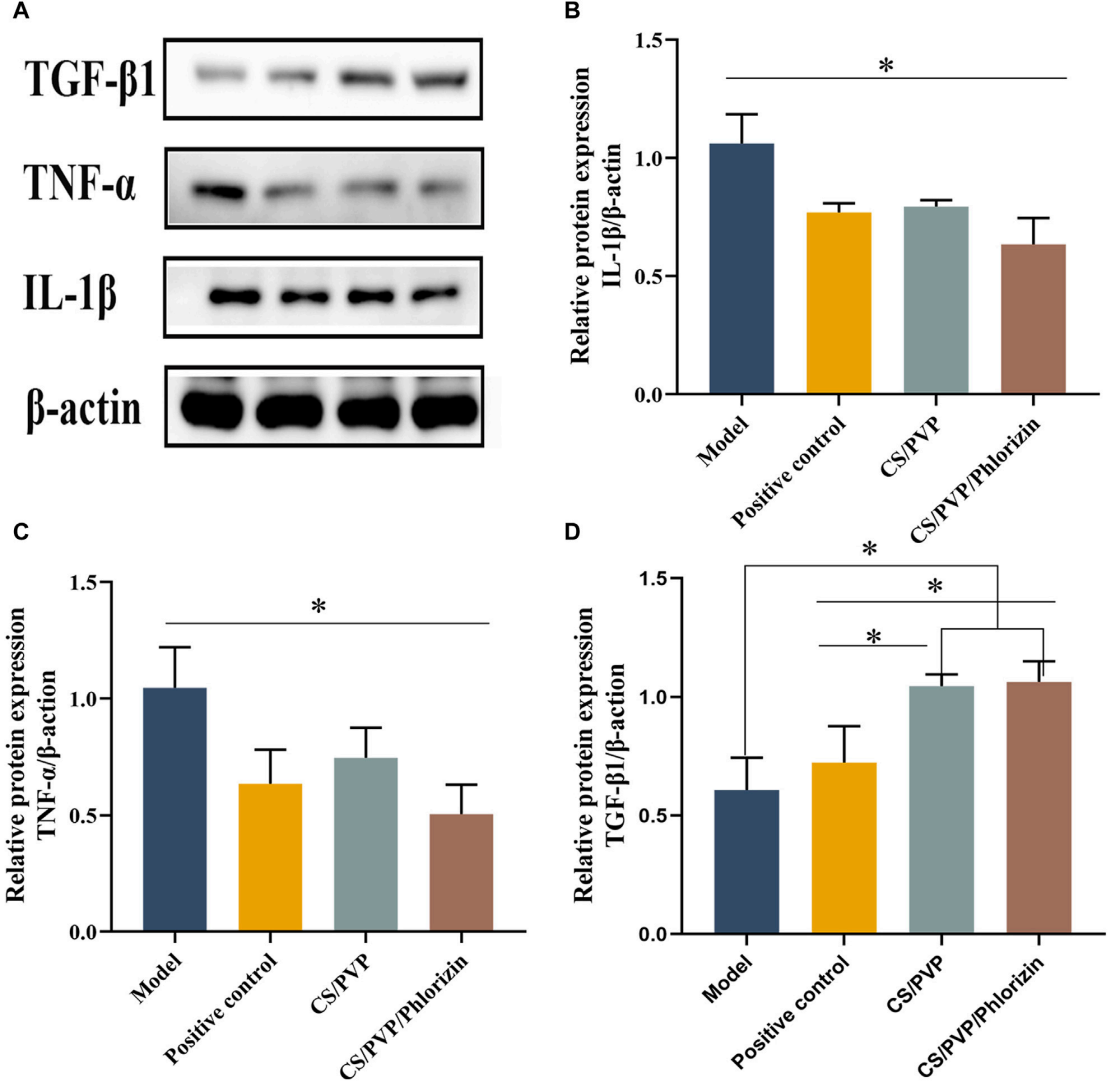
**(A)** Pictures of TGF-β1, TNF-α, and IL-1β protein bands; **(B)** Pictures of quantified gray values of IL-1β protein; **(C)** Pictures of quantified gray values of TNF-α protein; **(D)** Pictures of quantified gray values of TGF-β1 protein.

Following the onset of a burn, a good deal of leachate is present at the site of the burn, which is highly susceptible to infiltration by inflammatory cells, thus causing the onset of an inflammatory response (Liu, 2020). TNF-α and IL-1β, as the most important inflammatory factors in early inflammation, have a great influence on wound healing. There are different effects of different concentrations of TNF-α. Low concentrations of TNF-α activate T and B cells to release antibodies while also promoting phagocytosis by macrophages. However, at high concentrations, it enhances the inflammatory response causing the body to release more inflammatory factors, while causing damage to endothelial cells, contributing to increased vascular permeability and increased wound osmotic fluid thereby impeding wound healing (Peng and Huang, 2004). The results of the expression of TNF-α and IL-1β proteins in each group are shown in Figure 7 BC. TNF-α and IL-1β inflammatory proteins were significantly reduced in the group treated with Phlorizin fiber membrane compared with the scalded model group. The CS/PVP/Phlorizin group exhibited lower expression of inflammatory factors compared to the positive control group. In addition, we measured the expression of the growth factor TGF-β1 at the wound site in each group, and the results are shown in Figure 7D. CS/PVP and CS/PVP/Phlorizin significantly elevated the expression of TGF-β1 at the wound site compared with the model group.

## 4 Discussion

Scald wounds often cause large irregular skin defects and injuries, leading to disturbances in the patient’s internal environment, imbalance of the immune system, and infections, resulting in slow healing of the scald wounds and even further deepening of the wounds (Sahu et al., 2016). It is crucial to reduce scald wound infections and expedite the healing process for these patients. Research has indicated that incorporating natural actives into wound dressings can enhance wound healing (Li et al., 2022). In this study, we investigated the healing effects of phlorizin loaded into CS/PVP nanofiber membranes on burn wound injuries.

Nanofibrous membranes offer significant advantages in tissue repair due to their unique microstructure, which not only provides a suitable microenvironment for the wound site but also seals the active substances in the membrane to control their release for local delivery (Sun et al., 2022). Hydrophilicity is a necessary condition for the preparation of ideal nanofiber membranes, and it has been reported that nanofiber membranes with good hydrophilicity can rapidly absorb exudate from wound sites to promote wound repair. In this study, loading phlorizin into CS/PVP nanofiber membranes led to a decrease in the water contact angle and an enhancement of the hydrophilicity of the nanofiber membranes, which could be attributed to the increase in the diameter of the sparse nanofibers in the structure of the fiber membranes after loading phlorizin, which is more conducive to the absorption and permeation of water (Ding et al., 2024). At the same time, the CS/PVP/Phlorizin nanofiber membrane showed excellent antioxidant as well as antimicrobial activity, which could effectively inhibit the infection of the wound site and promote the wound healing process (Kumar et al., 2010). In addition, the nanofiber membrane has good blood compatibility and no cytotoxicity to meet the requirements of an ideal wound dressing (Shefa et al., 2017).

The process of scald wound repair involves various factors such as angiogenesis, cell proliferation, migration, and collagen formation at the wound site, all playing crucial roles in the healing process (Pelizzo et al., 2018; Zhang et al., 2019). Collagen, as an extracellular fibrous protein, promotes the formation of intracellular matrix and the repair of damaged cellular structures (Zhan et al., 2019). In the present study Masson staining results showed that CS/PVP/Plorizin nanofibrous membrane significantly promoted the formation of collagen at the wound site, which may be one of the reasons for its accelerated healing of scald wounds.

Growth factors are integral to the wound healing process. CD31 and VEGF, as vascular growth factors, can be evaluated for angiogenesis at the wound site (Torres et al., 2017). It was observed by immunohistochemical staining that the nanofibrous membrane group loaded with root bark glycosides significantly promoted the expression of CD31 and VEGF. Meanwhile, western blot results revealed that the nanofiber membrane not only decreased the expression of inflammatory factors at the wound site but also enhanced the expression of growth factor TGF-β1 and facilitated the accumulation of extracellular matrix, ultimately expediting the healing process of scalded wounds (Zhang et al., 2016; Zubair and Ahmad, 2019).

## 5 Conclusion

This study successfully loaded the natural antioxidant, phlorizin, into CS/PVP nanofiber membranes, demonstrating remarkable antioxidant, antimicrobial, and biocompatible properties. *In vivo* wound repair experiments revealed that the CS/PVP/Phlorizin nanofiber membrane effectively suppressed inflammatory reactions at the wound site by reducing the expression of inflammatory factors TNF-α and IL-1β. Furthermore, it facilitated blood vessel and collagen formation at the wound site, expediting the healing process of scalded wounds. The porous 3-dimensional mesh structure and hydrophilicity of the nanofiber membrane not only enhance air permeability but also address the challenge of administering drugs to irregular burn wounds. Overall, this innovative nanofiber membrane offers insights for developing new dressing for scald wound treatment.

## Data Availability

The original contributions presented in the study are included in the article/Supplementary Material, further inquiries can be directed to the corresponding authors.
