# Supplementary material for: Antibacterial and antioxidant phlorizin-loaded nanofiber film effectively promotes the healing of burn wounds
Source: Front Bioeng Biotechnol. 2024 Aug 5;12:1428988. doi: 10.3389/fbioe.2024.1428988 (PMC11330827; doi:10.3389/fbioe.2024.1428988)
Supplement: Supplementary file 1 [file Table1.docx]

| Antibodies | Factory owners |
| --- | --- |
| TGF-β1( DILUTION：1：1000 -1：5000 ) | Proteintech group |
| IL-1β ( DILUTION：1：2000-1：10000 ) | Proteintech group |
| TNF-α ( DILUTION：1：500-1：2000) | Proteintech group |
| β-actin (DILUTION：1：5000-1：50000) | Proteintech group |
| HRP coupled secondary antibodies (SA00001-1 1：2000-1：10000/SA00001-21：2000-1：10000) | Proteintech group |

Table 1：Experimental antibody sources and dilution ratios
