# Supplementary figures and images for: Antibacterial and antioxidant phlorizin-loaded nanofiber film effectively promotes the healing of burn wounds
Source: Front Bioeng Biotechnol. 2024 Aug 5;12:1428988. doi: 10.3389/fbioe.2024.1428988 (PMC11330827; doi:10.3389/fbioe.2024.1428988)

**1.Western Blot Original image**

**Repeat 1 Repeat 2 Pepeat 3**


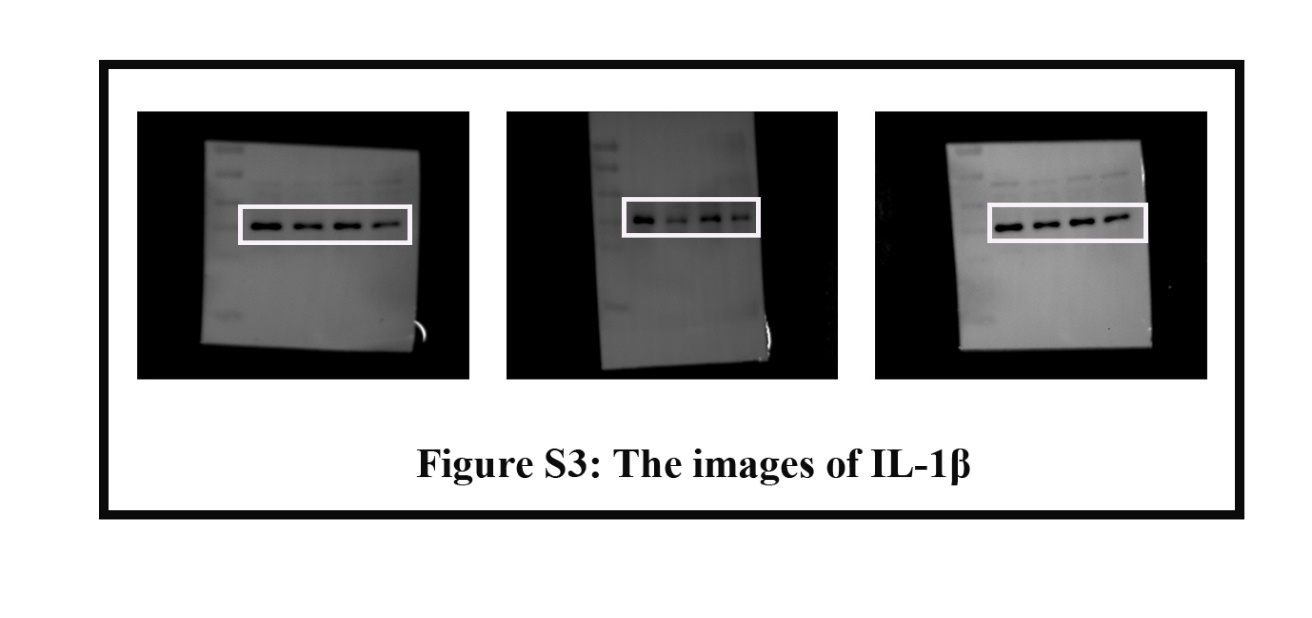

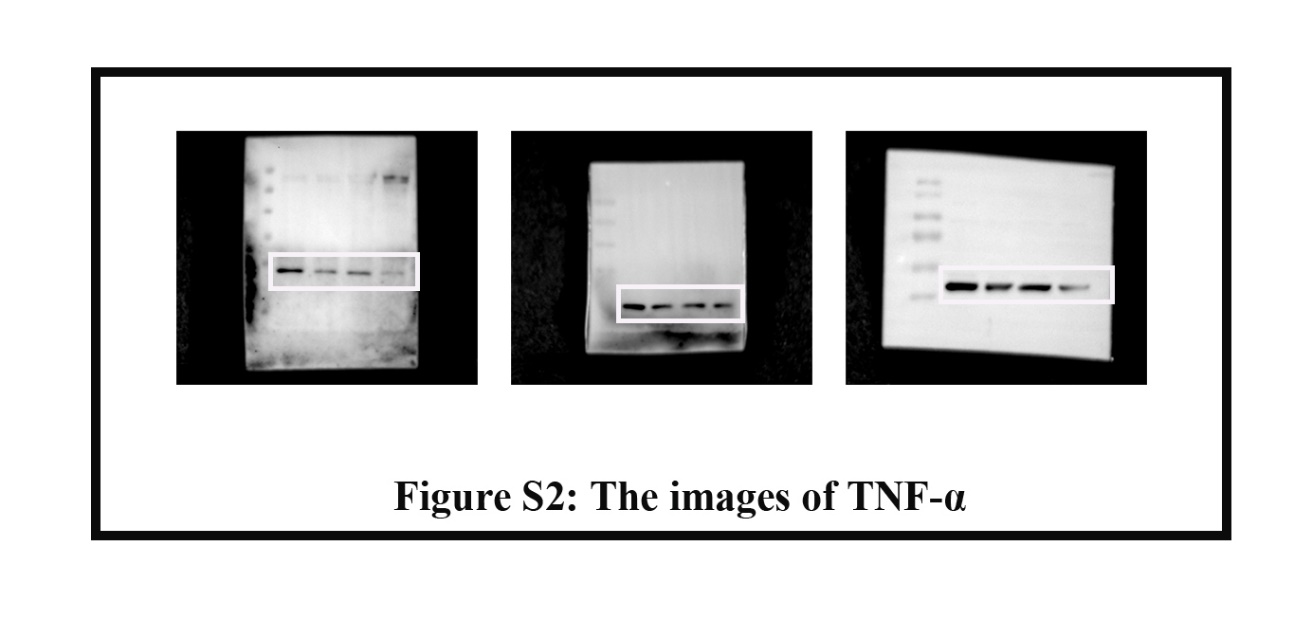

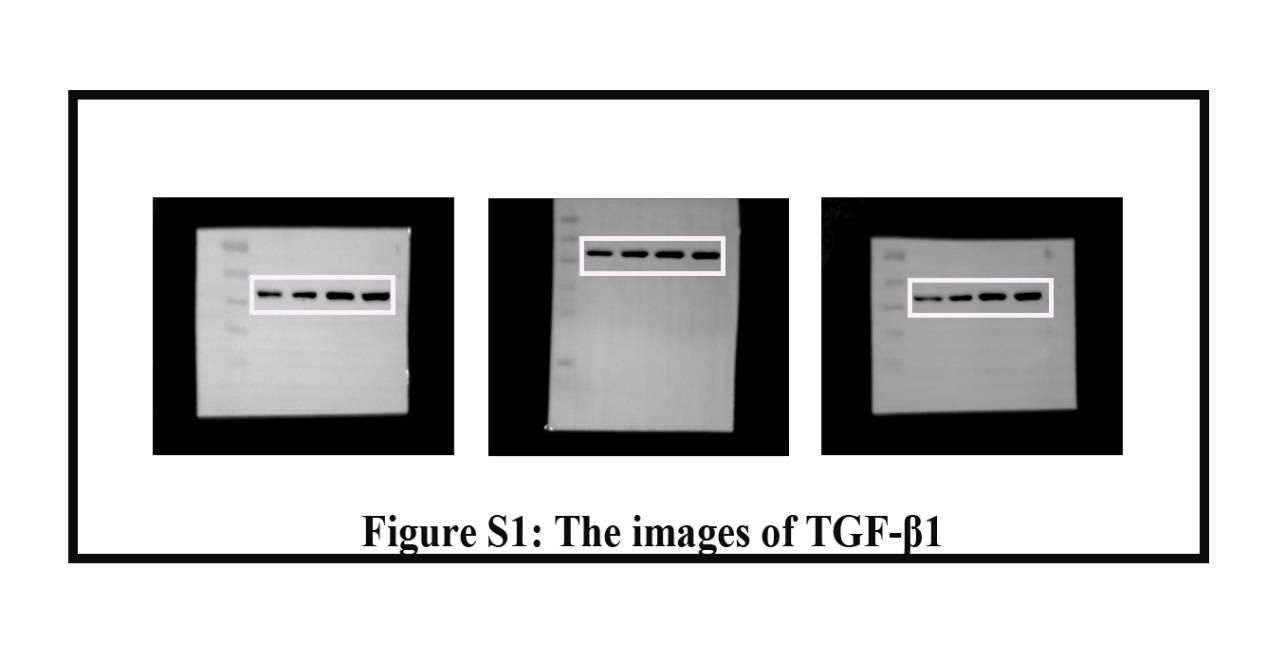


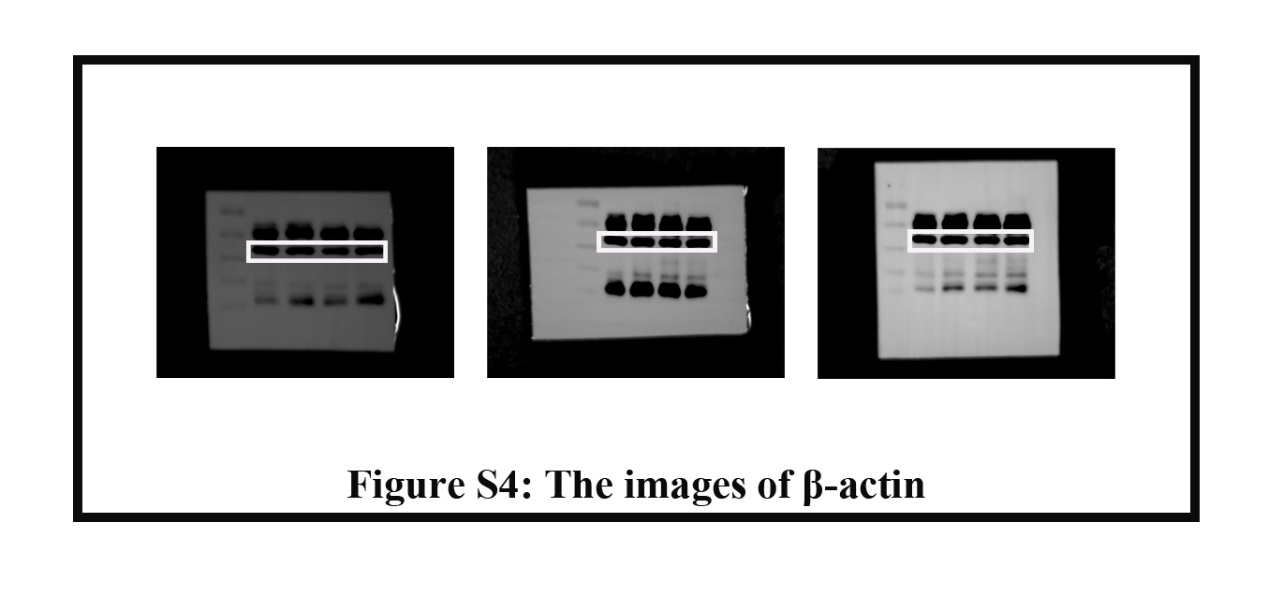

Supplement: Supplementary file 2 [file DataSheet1.docx]
